# Supplementary material for: Resolving the full spectrum of human genome variation using Linked-Reads
Source: Genome Res. 2019 Apr;29(4):635–45. doi: 10.1101/gr.234443.118 (PMC6442396; doi:10.1101/gr.234443.118)
Supplement: Supplemental Material [file supp_gr.234443.118_Supplemental_longranger-2.2.zip › longranger-2.2.tar.bz2!/lr2.2-public/lib/stan/cmdstan/stan/doc/api/html/dir_99762ae463b7309a47de4b14a14d0e0f.html]

Stan: src/stan/math/matrix Directory Reference


|  |  |
| --- | --- |
|  | Stan  2.5.0  probability, sampling & optimization |


- Main Page
- Related Pages
- Namespaces
- Classes
- Files

All Classes Namespaces Files Functions Variables Typedefs Enumerations Enumerator Friends Macros Pages

- src
- stan
- math
- matrix

matrix Directory Reference

|  |  |
| --- | --- |
| Directories | |
| directory | meta |
|  | |

|  |  |
| --- | --- |
| Files | |
| file | accumulator.hpp [code] |
|  | |
| file | add.hpp [code] |
|  | |
| file | append\_col.hpp [code] |
|  | |
| file | append\_row.hpp [code] |
|  | |
| file | array\_builder.hpp [code] |
|  | |
| file | assign.hpp [code] |
|  | |
| file | block.hpp [code] |
|  | |
| file | cholesky\_decompose.hpp [code] |
|  | |
| file | col.hpp [code] |
|  | |
| file | cols.hpp [code] |
|  | |
| file | columns\_dot\_product.hpp [code] |
|  | |
| file | columns\_dot\_self.hpp [code] |
|  | |
| file | common\_type.hpp [code] |
|  | |
| file | containers\_conversion.hpp [code] |
|  | |
| file | crossprod.hpp [code] |
|  | |
| file | cumulative\_sum.hpp [code] |
|  | |
| file | determinant.hpp [code] |
|  | |
| file | diag\_matrix.hpp [code] |
|  | |
| file | diag\_post\_multiply.hpp [code] |
|  | |
| file | diag\_pre\_multiply.hpp [code] |
|  | |
| file | diagonal.hpp [code] |
|  | |
| file | dims.hpp [code] |
|  | |
| file | distance.hpp [code] |
|  | |
| file | divide.hpp [code] |
|  | |
| file | dot\_product.hpp [code] |
|  | |
| file | dot\_self.hpp [code] |
|  | |
| file | Eigen.hpp [code] |
|  | |
| file | eigenvalues\_sym.hpp [code] |
|  | |
| file | eigenvectors\_sym.hpp [code] |
|  | |
| file | elt\_divide.hpp [code] |
|  | |
| file | elt\_multiply.hpp [code] |
|  | |
| file | exp.hpp [code] |
|  | |
| file | fill.hpp [code] |
|  | |
| file | get\_base1.hpp [code] |
|  | |
| file | get\_base1\_lhs.hpp [code] |
|  | |
| file | get\_lp.hpp [code] |
|  | |
| file | head.hpp [code] |
|  | |
| file | initialize.hpp [code] |
|  | |
| file | inverse.hpp [code] |
|  | |
| file | inverse\_spd.hpp [code] |
|  | |
| file | LDLT\_factor.hpp [code] |
|  | |
| file | log.hpp [code] |
|  | |
| file | log\_determinant.hpp [code] |
|  | |
| file | log\_determinant\_ldlt.hpp [code] |
|  | |
| file | log\_determinant\_spd.hpp [code] |
|  | |
| file | log\_softmax.hpp [code] |
|  | |
| file | log\_sum\_exp.hpp [code] |
|  | |
| file | max.hpp [code] |
|  | |
| file | mdivide\_left.hpp [code] |
|  | |
| file | mdivide\_left\_ldlt.hpp [code] |
|  | |
| file | mdivide\_left\_spd.hpp [code] |
|  | |
| file | mdivide\_left\_tri.hpp [code] |
|  | |
| file | mdivide\_left\_tri\_low.hpp [code] |
|  | |
| file | mdivide\_right.hpp [code] |
|  | |
| file | mdivide\_right\_ldlt.hpp [code] |
|  | |
| file | mdivide\_right\_spd.hpp [code] |
|  | |
| file | mdivide\_right\_tri.hpp [code] |
|  | |
| file | mdivide\_right\_tri\_low.hpp [code] |
|  | |
| file | mean.hpp [code] |
|  | |
| file | meta.hpp [code] |
|  | |
| file | min.hpp [code] |
|  | |
| file | minus.hpp [code] |
|  | |
| file | multiply.hpp [code] |
|  | |
| file | multiply\_lower\_tri\_self\_transpose.hpp [code] |
|  | |
| file | num\_elements.hpp [code] |
|  | |
| file | prod.hpp [code] |
|  | |
| file | promote\_common.hpp [code] |
|  | |
| file | promote\_scalar.hpp [code] |
|  | |
| file | promote\_scalar\_type.hpp [code] |
|  | |
| file | promoter.hpp [code] |
|  | |
| file | qr\_Q.hpp [code] |
|  | |
| file | qr\_R.hpp [code] |
|  | |
| file | quad\_form.hpp [code] |
|  | |
| file | quad\_form\_diag.hpp [code] |
|  | |
| file | rank.hpp [code] |
|  | |
| file | resize.hpp [code] |
|  | |
| file | row.hpp [code] |
|  | |
| file | rows.hpp [code] |
|  | |
| file | rows\_dot\_product.hpp [code] |
|  | |
| file | rows\_dot\_self.hpp [code] |
|  | |
| file | sd.hpp [code] |
|  | |
| file | segment.hpp [code] |
|  | |
| file | singular\_values.hpp [code] |
|  | |
| file | size.hpp [code] |
|  | |
| file | softmax.hpp [code] |
|  | |
| file | sort.hpp [code] |
|  | |
| file | sort\_indices.hpp [code] |
|  | |
| file | squared\_distance.hpp [code] |
|  | |
| file | stan\_print.hpp [code] |
|  | |
| file | sub\_col.hpp [code] |
|  | |
| file | sub\_row.hpp [code] |
|  | |
| file | subtract.hpp [code] |
|  | |
| file | sum.hpp [code] |
|  | |
| file | tail.hpp [code] |
|  | |
| file | tcrossprod.hpp [code] |
|  | |
| file | trace.hpp [code] |
|  | |
| file | trace\_gen\_inv\_quad\_form\_ldlt.hpp [code] |
|  | |
| file | trace\_gen\_quad\_form.hpp [code] |
|  | |
| file | trace\_inv\_quad\_form\_ldlt.hpp [code] |
|  | |
| file | trace\_quad\_form.hpp [code] |
|  | |
| file | transpose.hpp [code] |
|  | |
| file | typedefs.hpp [code] |
|  | |
| file | variance.hpp [code] |
|  | |


---

[ Stan Home Page ]

*© 2011–2014,
Stan Development Team.*
